# Control of root-knot nematode disease in *Aucklandia lappa* Decne. by endophytic *Serratia liquefaciens* ML4

**DOI:** 10.3389/fpls.2026.1895609

**Published:** 2026-07-27

**Authors:** Yunxia Li, Yanmei Yang, Li Luo, Fuxiang Liu, Qiankun Li, Bokai Bi, Hanyang Yao, Luoli Shi, Xiujun Li, Xianqi Hu

**Affiliations:** 1 College of Plant Protection, Yunnan Agricultural University/State Key Laboratory of Conservation and Utilization of Biological Resources, Kunming, China; 2 Yunnan Hengyuan Detection Technology Co., Ltd., Kunming, China; 3 Kunming Baoteng Biochemical Technology Co., Ltd., Kunming, China

**Keywords:** 1-octen-3-one, *Aucklandia lappa* Decne., biocontrol, *Meloidogyne hapla*, volatile organic compounds

## Abstract

**Introduction:**

*Aucklandia lappa* Decne. is a medicinal plant. In recent years, *Meloidogyne hapla* has been found to cause severe damage to *A. lappa*. Given the medicinal nature of *A. lappa*, disease control must prioritize safety concerns such as pesticide residues. Therefore, environmentally friendly and highly safe biological control measures are of considerable significance for managing root-knot nematode disease in this plant, and endophytic bacteria represent a valuable source of biocontrol resources.

**Methods:**

A strain of *Serratia liquefaciens* ML4 with the ability to control root-knot nematode disease was screened from endophytic bacteria of *A. lappa*. The nematicidal activity of its fermentation broth was evaluated through in vitro assays, pot experiments, and field micro-plot trials. The composition of the fermentation broth was analyzed by headspace solid-phase microextraction coupled with gas chromatography-mass spectrometry (HS-SPME-GC-MS).

**Results:**

The 2-fold dilution of the fermentation broth achieved a corrected mortality rate of 95.82% against J2s of *M. hapla* after 24 h of treatment. The egg hatching rate was inhibited by 99.51%, the pot control efficacy was 57.14%, and the field micro-plot test control efficacy was 62.79%. At the same time, it promoted the growth of *A. lappa*. Active volatile organic compounds (VOCs), including 1-octen-3-one, 6-methyl-5-hepten-2-one, dimethyl trisulfide, 2-phenylethanol, dimethyl disulfide, and 2-isobutyl-3-methylpyrazine, were screened from the fermentation broth and demonstrated nematicidal activity against J2s of *M. hapla*. Among them, 1-octen-3-one exhibited a fumigation LC_50_ of 5.45 × 10_-3_ µL/L and an aqueous contact LC_50_ of 5.79 µL/L against J2s. The pot experiment showed that at a concentration of 1 µL/L, 1-octen-3-one inhibited gall formation by 79.66%.

**Conclusion:**

*S. liquefaciens* ML4 can effectively control root-knot nematode disease on *A. lappa* and promote plant growth. The VOCs in its fermentation broth are the key active ingredients responsible for nematicidal activity and disease control, indicating this strain is promising for biocontrol of root-knot nematodes.

## Highlights

- The endophytic strain *Serratia liquefaciens* ML4, isolated from *Aucklandia lappa*, possesses dual functions: controlling root-knot nematode disease and promoting plant growth.
- The nematicidal mechanism of strain ML4 primarily depends on the production of secondary metabolites.
- This study is the first to report that the volatile organic compounds 1−octen−3−one and 6-methyl-5-hepten-2-one, identified in ML4 fermentation broth, exhibit nematicidal activity against *Meloidogyne hapla*.

## 1 Introduction

Root-knot nematode (*Meloidogyne* spp.) is an important pathogen affecting many crops, including food crops, vegetables, fruit trees, and medicinal plants. The host range of *Meloidogyne* spp. exceeds 3000 plant species, often resulting in serious yield losses (Abad et al., 2003). Root-knot nematodes infect plants to form giant cells, disrupt root structure and function, weaken the ability of plants to absorb water and nutrients from soil, and consequently affect plant growth and development (Escobar et al., 2015). In addition, wounds caused by root-knot nematode infection facilitate the entry of soil-borne pathogens (e.g., *Fusarium*), leading to complex diseases and aggravated damage (Keinath and Agudelo, 2018). *Aucklandia lappa* Decne. is a perennial herb of the Asteraceae family. Its dried roots have been used as medicine for more than 2,000 years (Huang et al., 2022). Currently, it is mainly cultivated in Yunnan, Sichuan, Guizhou, Guangxi, and other provinces or autonomous regions of China (Xue et al., 2020). It is an important medicinal material widely used in traditional Chinese medicine and is officially recorded in the Chinese Pharmacopeia (Huang et al., 2021). Since the establishment of industrial bases in Yunnan and other regions in 2014, the planting scale of *A. lappa* has expanded, and the economic benefits have been gradually improved. In 2024, the national sales volume exceeded 8300 tons (Chen et al., 2025). Modern pharmacological studies have demonstrated that *A. lappa* exerts multiple effects, including promoting gastrointestinal motility, dilating bronchial smooth muscle, and exhibiting antibacterial, hypoglycemic, and anti-tumor properties (Song et al., 2021; Zhuang et al., 2021; Song et al., 2022; Zhang et al., 2025). However, with the development of intensive and large-scale planting of *A. lappa*, various pests and diseases have become increasingly prevalent, among which root-knot nematode disease is particularly serious. At present, it is clear that the pathogen is *Meloidogyne hapla*. Given the particularity of *A. lappa* as a medicinal plant, its disease prevention and control should place special emphasis on safety issues, such as pesticide residues. Therefore, environmentally friendly and safe biological control measures are of great significance in the prevention and control of root-knot nematode disease.

The prevention and treatment of root-knot nematode disease is still dominated by chemical control. Common chemical nematicides include abamectin, fosthiazate, and fluopyram, among others (Zhang et al., 2022). Single chemical control not only poses a potential risk to the ecological environment and public health, but continuous use of chemical nematicides may also lead to pesticide resistance in nematodes. Rotation and utilization of resistant cultivars are important control measures. However, their application is limited by the wide host range of root-knot nematodes, difficulties in rotation implementation, and possible loss of cultivar resistance (Mata et al., 2025). Resistant cultivars of medicinal plants are currently difficult to find. As an environmentally friendly strategy for root-knot nematode disease prevention and control, biological control has the advantages of a safe ecological environment, a diverse mechanism of action, and low susceptibility to pesticide resistance. It can effectively reduce the use of chemical pesticides and fertilizers, which aligns with the concept of sustainable development (Raymaekers et al., 2020). Therefore, mining biocontrol resources and finding more biocontrol strains will remain research hotspots for a long time to come.

Previous studies have shown that most biocontrol bacteria possess multifunctional benefits, can effectively control pests and diseases, and can promote plant growth (Bhat et al., 2023). For example, plant growth-promoting rhizobacteria (PGPR) such as *Bacillus* (Antil et al., 2022), *Pseudomonas* (Zhao et al., 2018), and *Serratia* (Xiang et al., 2018) have both disease prevention and growth promotion functions. These biocontrol bacteria can solubilize phosphorus, solubilize potassium, fix nitrogen, and provide more available nutrients for plant growth (Jooste et al., 2019; Alvarado et al., 2025). They can produce siderophores, which enhance iron uptake by plants through chelation of iron in the environment (Timofeeva et al., 2022); plant growth regulators such as indole-3-acetic acid (IAA) can be synthesized to stimulate root development and promote nutrient absorption (Duca and Glick, 2020). Biocontrol fungi such as *Paecilomyces lilacinus* (Monjil and Ahmed, 2019) and *Pochonia chlamydosporia* (Parveen et al., 2025) can also control root-knot nematode disease and promote crop growth.

Biocontrol bacteria are generally considered to have diverse mechanisms of action, which may reduce the risk of target organisms developing pesticide resistance. First, they can directly antagonize root-knot nematodes by producing antibacterial secondary metabolites and proteins and by forming biofilms (Paiva et al., 2013; Proenca et al., 2019). Second, they can express enzymes such as proteases, lipases, and chitinases to degrade nematode body wall components (Zhang et al., 2018). Third, they can produce catalase and other antioxidant enzymes, which help plants cope with oxidative stress and enhance plant resistance (Khanna et al., 2019). These characteristics and activities enable biocontrol bacteria to improve plant growth and nutrient utilization, thereby strengthening the natural defense mechanisms of plants, inducing resistance to various biotic and abiotic stresses, which is of considerable value for sustainable agriculture (Farrar et al., 2014; Pardo-Díaz et al., 2021).

Driven by the concepts of green, safe, and sustainable development, this study investigated a strain of *Serratia liquefaciens* ML4 (Chinese Invention Patent ZL 2024 1 1328274.3), which was previously isolated from healthy roots of *A. lappa* in our laboratory. Through *in vitro* nematicidal activity, pot experiments, and field micro-plot experiments, the nematicidal activity of ML4 against second-stage juveniles (J2s) of root-knot nematodes and its biocontrol efficacy against root-knot nematode disease were determined. Furthermore, analysis of the fermentation broth components revealed that volatile organic compounds (VOCs), including 1-octen-3-one (Chinese Invention Patent ZL 2026 1 0055634.X), 6-methyl-5-hepten-2-one, and dimethyl trisulfide, exhibited pronounced effects in controlling the disease. This study thus elucidates the intrinsic mechanisms underlying the nematicidal activity and disease control mediated by ML4.

## 2 Materials and methods

### 2.1 Acquisition of strains and nematodes

*S. liquefaciens* ML4 was isolated from healthy roots of *A. lappa* grown in Ludian Township, Yulong County, Lijiang City, Yunnan Province, China. The strain was preserved in the Laboratory of Plant Nematology, College of Plant Protection, Yunnan Agricultural University, and deposited in the China General Microbiological Culture Collection Center (CGMCC) (No. 3, Yard 1, Beichen West Road, Chaoyang District, Beijing, China) under accession number CGMCC No. 30599. *M. hapla* was isolated from *A. lappa* plants infected with root-knot nematode.

### 2.2 Preparation of ML4 fermentation broth and collection of supernatant

After activation of strain ML4, a single colony was inoculated into 100 mL of LB liquid medium in a 250 mL Erlenmeyer flask. The culture was incubated at 32 °C and 180 rpm for 24 h to obtain the seed culture. The seed culture was then transferred into fresh LB liquid medium at a 5% (v/v) inoculation rate, and the culture was incubated at 32 °C and 180 rpm for 24–120 h (Zaheer et al., 2016). The resulting fermentation broth (1.25 × 10^11^ cfu/mL) was centrifuged at 12,000 rpm for 10 min, and the supernatant was collected. The supernatant was subsequently diluted 2.5-, 5-, 10-, 25-, and 50-fold for use in subsequent experiments.

### 2.3 Nematicidal activity, egg hatching inhibition, and chemotaxis of fermentation supernatant against *M. hapla*

Nematicidal activity against *M. hapla* J2s: 12-well plates were used. Each well contained 500 μL of serially diluted fermentation supernatant, 400 μL of nematode suspension (approx. 150 J2s), and 100 μL of 2% (m/v) streptomycin sulfate. The final dilutions of the fermentation supernatant were 2-, 5-, 10-, 20-, 50-, and 100-fold. LB liquid medium was used as a control. Each treatment was replicated six times. The plates were incubated at 25 °C, and mortality was recorded after 24 and 48 h. Both the mortality rate and the corrected mortality rate were calculated.

Mortality (%) = number of deaths/total number × 100% (1).

Corrected mortality (%) = (mortality of treatment group - mortality of control group)/(1-mortality of control group) × 100% (Liu et al., 2022) (2).

Egg hatching inhibition: 12-well plates were used. Each well was loaded with 500 μL of fermentation supernatant and 500 μL of nematode egg suspension (approx. 800 eggs). The working concentrations were the same as those used in the nematicidal activity assay against *M. hapla* J2s, and LB liquid medium was used as the control. Each treatment was replicated four times. After incubation at 25 °C in darkness for 72 h, the number of hatched juveniles, the total number of eggs, the hatching rate, and the hatching inhibition rate were calculated.

Hatching rate (%)= hatching number/total number of eggs × 100%(3).

Hatching inhibition rate (%) = (control hatching rate - treatment hatching rate)/control hatching rate × 100% (Mishra et al., 2017) (4).

Nematode chemotaxis experiment: according to the method (Cheng et al., 2017), 12 h observation statistics were collected, and the chemotaxis index (C.I.) was calculated. When 0 < C.I. <1, it is attractive. When -1 < C.I.<0, it is a repellent; when C.I. = 0, there was no significant effect on chemotaxis.

C.I.= (number of nematodes in the test area - number of nematodes in the control area)/(number of nematodes in the test area + number of nematodes in the control area)(5).

Repellent rate (%)=number of nematodes in the control area/total number of nematodes × 100%(6).

### 2.4 Nematicidal activity of fermentation broth volatiles against *M. hapla* J2s

Three-compartment Petri dishes were used in this experiment. In the treatment group, 8 mL of undiluted fermentation broth was added to compartment A, whereas 8 mL of LB liquid medium was added to compartment A of the control group. A 200 μL aliquot of nematode suspension containing approximately 150 J2s was placed in compartment B. Each treatment was replicated six times. After sealing, the dishes were incubated at 25 °C, and the corrected mortality of J2s was recorded 24 h post-treatment (Song et al., 2024).

### 2.5 Field micro-plot experiment

The experimental base was located in Xinzhu Village, Ludian Township, Yulong County, Lijiang City, Yunnan Province, with an area of approximately 1334 m^2^. The initial population density of root-knot nematode J2s detected in soil samples was approximately 350/100 cm^3^. One-year-old seedlings of the test plant *A. lappa* were cultivated by local villagers.

The fermentation broth for the field trial was obtained from a separate batch prepared under the same conditions as described in Section 2.2, with a final concentration of 1.27 × 10¹¹ cfu/mL. This broth was tested at seven concentrations: undiluted and 2-, 5-, 10-, 20-, 50-, and 100-fold dilutions. Each *A. lappa* plant was drenched with 50 mL of the fermentation broth at the corresponding concentration. Two control groups were established: 10% fosthiazate granules (Foshan Yinghui Crop Science Co., Ltd.) were applied at 0.3 g per plant, and 50 mL of water was used to irrigate the roots. Two application schedules were established: (i) a single application at transplanting (0 d), and (ii) two applications, one at transplanting and another 30 days after transplanting (0&30 d). The field experiment comprised a total of 17 treatments. Each treatment consisted of three plots, with at least 30 A*. lappa* plants planted per plot. The plots were randomly assigned to the treatments. For statistical analysis, data from each micro−plot were first averaged to obtain a single value per replicate; thus, the micro−plot (not the individual plant) served as the experimental unit (n = 3 per treatment). Differences among treatments were evaluated using one-way analysis of variance (ANOVA), followed by Fisher’s least significant difference (LSD) test at α = 0.05.

The SPAD (Soil-Plant Analysis Development) value and disease index of *A. lappa* were recorded at 60 days after transplanting, and the relative control efficacy was calculated. At 90 days after transplanting, the fresh weights of both aboveground and underground parts of *A. lappa* were measured, and the relative control efficacy was calculated. Yield was determined at harvest, and the relative control efficacy was also calculated. The disease classification standard referred to the ten-level classification standard reported by Bridge and Page (Bridge and Page, 1980).

Disease index =∑ (number of diseased plants at all levels × series) × 100/(total number of investigations × highest value) (7), Relative control efficacy = (disease index of control group - disease index of treatment group)/disease index of control group × 100% (Tian et al., 2022) (8).

### 2.6 Pot experiment

The fermentation broth concentrations and controls used in the pot experiment were identical to those in the field experiment. Three application schedules were established: (i) a single application at transplanting (0 d); (ii) two applications, one at transplanting and another 30 days after transplanting (0 & 30 d); and (iii) a single application at 30 days after transplanting (30 d). A total of 27 treatments were included, each with six replicate pots. Field soil naturally infested with nematodes was collected, mixed, and used to fill pots (16 cm diameter × 22 cm height). *A. lappa* seedlings were transplanted into each pot, and each pot was inoculated with 1,000 J2s. The pots were buried in the field to maintain soil temperature and humidity consistent with the surrounding environment. Disease index, plant height, stem diameter, and aboveground and underground fresh weights were measured at 60 days after transplanting. For statistical analysis, a single observed value was obtained for each replicate, and each pot served as an experimental unit (n = 6 per treatment). Differences among treatments were evaluated using one-way ANOVA, followed by Fisher’s LSD test at α = 0.05.

### 2.7 Analysis of VOCs in the fermentation broth of strain ML4

Identification of volatile substances in the fermentation broth of strain ML4 was performed by Shanghai Majorbio Bio-Pharm Technology Co., Ltd. (Shanghai, China) using headspace solid-phase microextraction coupled with gas chromatography-mass spectrometry (HS-SPME-GC-MS). The VOCs were classified, and their odor contributions were analyzed on the Majorbio cloud platform.

### 2.8 Nematicidal activity assay of VOCs from fermentation broth against *M. hapla*

The compounds 1-octen-3-one (95% purity), 6-methyl-5-hepten-2-one (98% purity), dimethyl trisulfide (98% purity), dimethyl disulfide (98% purity), phenylethyl alcohol (99% purity), and 2-isobutyl-3-methylpyrazine (98% purity) were purchased from Rhawn Reagent (Shanghai Yien Chemical Technology Co., Ltd., Shanghai, China).

Fumigation nematicidal activity of VOCs. Each well of a 12-well plate was loaded with 500 μL of *M. hapla* J2 suspension (approx. 150 J2s). The plate was placed in a 3.5 L sealed chamber, into which 7 μL of neat VOCs was directly injected to achieve a final concentration of 2 μL/L. The control group received no VOCs. Each treatment had six replicates. The chamber was incubated at 26 °C in the dark for 24 h. The total and dead J2s were counted, and the mortality and corrected mortality were calculated.

### 2.9 Determination of LC_50_ of 1-octen-3-one against *M. hapla* J2s by contact and fumigation assays

Contact activity: A 12−well plate was used. Into each well, 500 μL of 1-octen-3-one aqueous solution and 500 μL of *M. hapla* J2 suspension (approx. 150 J2s) were added to achieve final concentrations of 1-octen-3-one at 2, 4, 6, 8, 10, and 12 μL/L. Sterile water served as the control. Each treatment was replicated six times. The plate was incubated at 26 °C for 12 h. Then, the total number of nematodes and the number of dead J2s in each well were counted, and the mortality rate and corrected mortality rate were calculated.

Fumigation activity: A 12-well plate was used, and 500 μL of *M. hapla* J2 suspension (approx. 150 J2s) was added to each well. The plate was placed in a sealed chamber. Neat 1−octen−3−one was introduced into the chamber to achieve vapor concentrations of 1 × 10^−2^, 5 × 10^−3^, 2.5 × 10^−3^, 1.25 × 10^−3^, and 0.625 × 10^−3^ μL/L (v/v), respectively. The control group received no 1−octen−3−one. Each treatment was replicated six times. The chamber was maintained at 26 °C in darkness for 12 h. Subsequently, the total number of nematodes and the number of dead J2s in each well were counted, and the mortality rate and corrected mortality rate were calculated.

The median lethal concentration (LC_50_) was calculated by curve fitting using GraphPad Prism 10.0 software.

### 2.10 Pot experiment of 1-octen-3-one against root-knot nematode disease

Cucumber plants (cv. ‘Jinyan No. 4’) were grown in plastic pots measuring 10 cm in diameter and 14 cm in height, with one plant per pot. The potting mixture consisted of a 1:1 (v/v) ratio of substrate to field soil, and each pot was filled with 1 L of the mixed soil. When the cucumber seedlings reached the four-leaf stage, each pot was inoculated with 1,200 J2s of *M. javanica*. Simultaneously, 1-octen-3-one was added to each pot at volumes of 0.005, 0.01, 0.1, and 1 μL. The pots were immediately sealed with resealable plastic bags to achieve volatile concentrations of 5 × 10^-3^, 1 × 10^-2^, 1 × 10^-1^, and 1 μL/L in the soil. After 3 days of sealing, the bags were removed. A control group was set up without the addition of 1-octen-3-one. Each treatment was replicated five times. The plants were grown for 30 days, after which they were harvested. The number of root galls per root system was counted, and the gall inhibition rate was calculated. Although *M. hapla* and *A. lappa* are naturally adapted to cooler climates, they did not perform reproducibly under our greenhouse conditions. We therefore adopted the *M. javanica*–cucumber pathosystem, which was well−suited to our greenhouse environment and enabled consistent, repeatable infection assays. The selection of *M. javanica* as a representative species was further justified by the previously demonstrated broad−spectrum nematicidal activity of 1−octen−3−one against multiple plant−parasitic nematodes, including *M. incognita, M. hapla, and M. enterolobii* (patent No. ZL 2026 1 0055634.X).

Root knot inhibition rate(%) =(1 - treatment root knot number/control root knot number) × 100% (Tian et al., 2022). (9).

### 2.11 Data analysis

**Figure 1:**
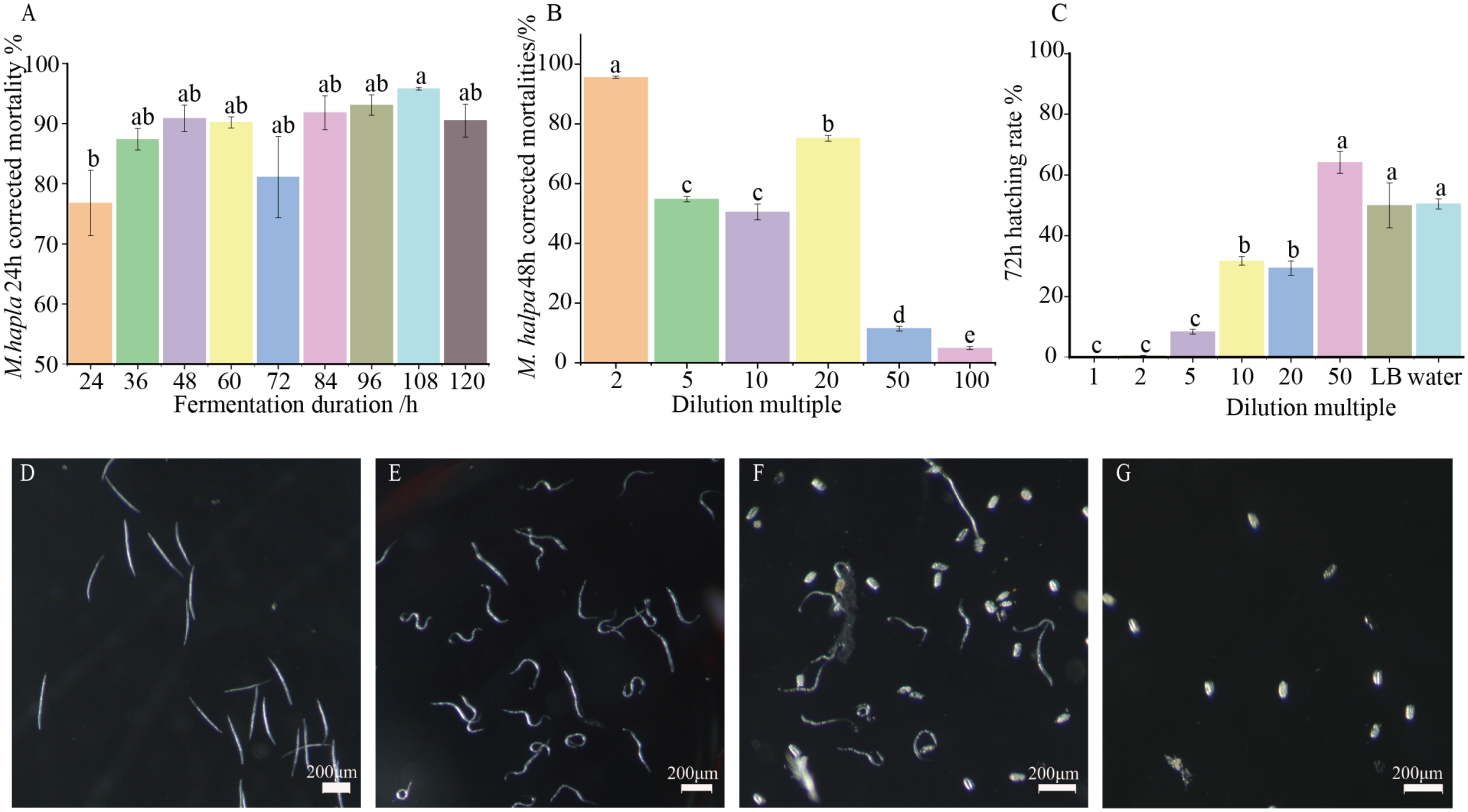
*In vitro* nematicidal activity of fermentation broth of *S. liquefaciens* ML4 against *M. hapla*. **(A)** Nematicidal activity of 2-fold diluted fermentation broths obtained at different fermentation times against *M hapla* after 24 h of exposure. **(B)** Nematicidal activity of the 108 h fermentation broth at different dilutions after 48 h of exposure. **(C)** Egg hatching rates after 72 h of exposure to different dilutions of the 108 h fermentation broth. **(D)** Mortality status of *M. hapla* J2s after exposure to the fermentation broth. **(E)** *M. hapla* J2s in LB liquid medium after 48 h (control). **(F)** Egg hatching of *M. hapla* in LB liquid medium. **(G)** Unhatched eggs of *M. hapla* in the fermentation broth. Different letters indicate significant differences at P<0.05 based on Tukey’s HSD test. In the picture, 1: the undiluted fermentation supernatant; 2, 5, 10, 20, 50, 100: 2-, 5-, 10-, 50-, 100-fold diluted fermentation supernatant; LB: LB liquid medium control; water: sterile water control.

**Figure 2:**
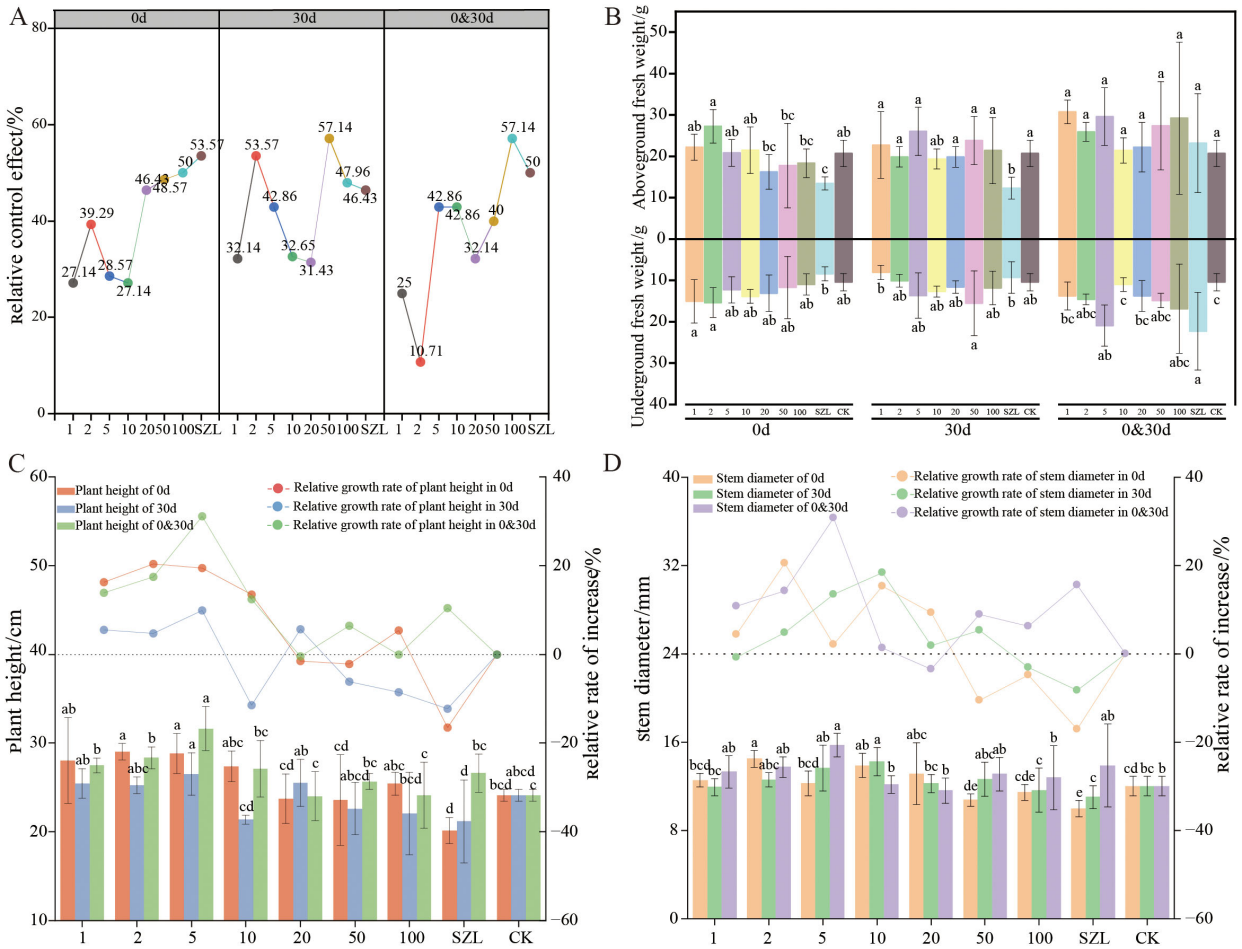
Pot experiment results for the control of *M. hapla* on *A. lappa* by *S. liquefaciens* ML4 at 60 days after treatment. **(A)** Relative control efficacy of fermentation broth applied at 0 d, 30 d, and 0&30 d. **(B)** Aboveground and underground fresh weight. **(C)** Plant height and plant height growth rate. **(D)** Stem diameter and stem diameter growth rate. In the figure 0 d: a single application at transplanting; 30 d: a single application at 30 days after transplanting; 0&30 d: two applications, one at transplanting and another at 30 days after transplanting; 1: the undiluted fermentation broth; 2, 5, 10, 20, 50, 100: 2-, 5-, 10-, 50-, 100-fold diluted fermentation broth; SZL: application of 10% fosthiazate 0.3 g, CK: water control. Different letters indicate significant differences at P<0.05 based on Fisher’s LSD test.

**Figure 3:**
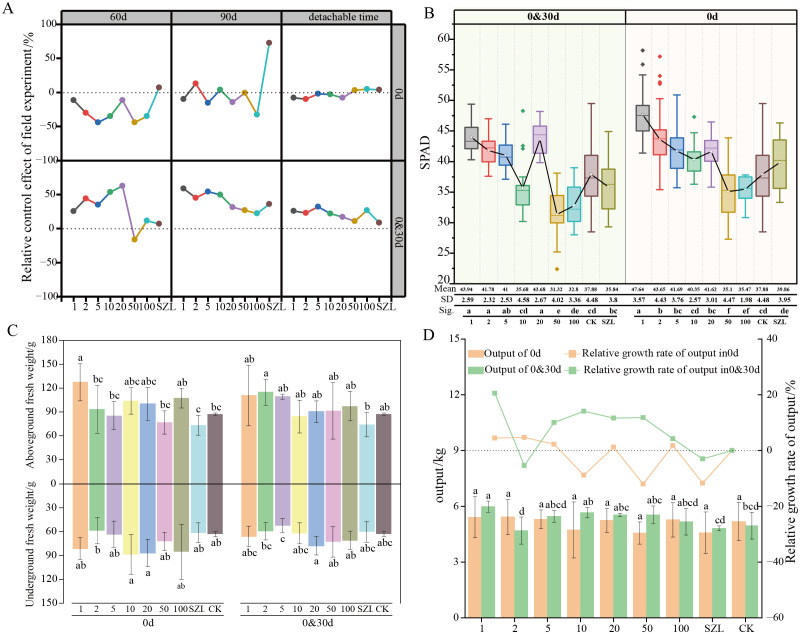
Field micro-plot experiment results of *S. liquefaciens* against *M. hapla*. **(A)** Relative control efficacy at 60 d, 90 d, and harvest following fermentation broth application at 0 d and 0&30 d **(B)** SPAD values of each treatment at 60 days after transplanting. **(C)** Aboveground and underground biomass at 90 d after transplanting, following fermentation broth application at 0 d and 0&30 d **(D)** Yield at harvest following fermentation broth application at 0 d and 0&30 d.

Data analysis and visualization were performed using SPSS, Origin, and GraphPad Prism software. For **Figure 1**, Tukey’s test was used, and for **Figures 2**, **3**, Fisher’s LSD test was used. The LC_50_ of 1-octen-3-one against J2s was determined by nonlinear regression of the log(dose)–response curve using GraphPad Prism 10.0. (Talavera-Rubia et al., 2020; Yang et al., 2025).

## 3 Results

### 3.1 *In vitro* nematicidal activity

The fermentation broth of strain ML4 cultured in LB liquid medium exhibited significant nematicidal, egg-hatching inhibitory, and repellent effects against *M. hapla* J2s.

When the fermentation broth was applied against *M. hapla* for 24 h, the nematicidal activity was significantly influenced by fermentation time. A fermentation period of 36 h or longer resulted in significantly higher activity compared with 24 h of fermentation (76.82%). The optimal fermentation time was 108 h, which yielded a corrected mortality of 95.82% (**Figure 1A**). The 108 h fermentation broth was further tested at different dilutions. After 48 h of exposure, the corrected mortalities against *M. hapla* J2s were 95.69% at 2-fold dilution and 75.26% at 20-fold dilution (**Figure 1B**).

The undiluted 108 h fermentation broth achieved an egg hatching inhibition rate of 99.51%. After 72 h of treatment, the hatching rates of eggs exposed to the undiluted fermentation broth and its 2-, 5-, 10-, and 20-fold dilutions were 0.24%, 0.46%, 8.34%, 31.70%, and 29.31%, respectively. These values were significantly lower than the hatching rates observed in the LB liquid medium, sterile water, and 50-fold dilution controls, which were 50.01%, 50.50%, and 64.18%, respectively (**Figure 1C**).

In addition to its contact activity and ovicidal activity, the undiluted fermentation broth also exhibited potent fumigant toxicity and repellent effects against J2s. The undiluted fermentation broth exhibited a corrected mortality of 96.43% against *M. hapla* J2s after 24 h of exposure to its VOCs. After treatment with the fermentation broth for 12 h, the repellency rate against *M. hapla* J2s reached 96.20%, and the chemotaxis index (C.I.) was −0.92.

### 3.2 Efficacy in pot experiment

Application of strain ML4 fermentation broth in pot experiments effectively controlled *M. hapla* disease on *A. lappa* and promoted plant growth.

The relative control efficacy evaluated at 60 days post−transplanting in the pot experiment (**Figure 2A**) showed that both single and double applications of different dilutions of the fermentation broth effectively controlled root-knot nematode disease on *A. lappa*. For a single application at 0 d, the relative control efficacy ranged from 27.14% to 50%, with the 100-fold dilution achieving the highest efficacy (50%). For a single application at 30 d, the relative control efficacy ranged from 31.42 to 57.14%, with the 50-fold dilution showing the best efficacy (57.14%). For two applications (0&30 d), the relative control efficacy ranged from 10.71% to 57.14%, and the 100-fold dilution exhibited the highest efficacy (57.14%).

Biomass investigation at 60 days post-transplanting (**Figure 2B**) showed that the application of fermentation broth increased the biomass of *A. lappa*. Two applications of the fermentation broth promoted the accumulation of both aboveground and underground biomass more effectively than a single application, and higher concentrations were more favorable for aboveground biomass accumulation than lower concentrations. When applied at 0 d, the undiluted fermentation broth (15.05 g) and the 20-fold dilution (15.35 g) significantly increased underground biomass compared with the positive control (10% fosthiazate granules at 8.43 g) (p< 0.05). A single application at 30 d of the 50-fold dilution (15.53 g) resulted in a significantly higher underground fresh weight than that of the fosthiazate treatment (9.28 g). For two applications (0&30 d), all tested dilutions increased underground biomass accumulation; the 5-fold dilution significantly increased the underground fresh weight (20.93 g) compared with the control (10.43 g).

Plant height measurements showed that a single application at 0 d of the 2-fold (relative growth rate: 20.31%, 29.03 cm) and 5-fold (19.48%,28.83cm) dilutions, as well as two applications at 0 & 30 d of the undiluted fermentation broth (13.89%, 27.48 cm), 2-fold (17.51%, 28.35 cm), and 5-fold (31.09%, 31.63 cm) dilutions, significantly increased the plant height of *A. lappa* (p < 0.05) (**Figure 2C**).

Stem diameter measurements showed that, compared with the control (12.02 mm), single applications at 0 d of the 2-fold (20.57%,14.49 mm) and 10-fold (15.39%,13.87 mm) dilutions, a single application at 30 d of the 10-fold dilution (18.45%,14.24 mm), and two applications at 0 and 30 d of the 5−fold dilution (30.84%,15.73 mm) significantly increased the stem diameter of *A. lappa* (p < 0.05) (**Figure 2D**).

### 3.3 Efficacy in field micro-plot experiment

#### 3.3.1 Relative control efficacy

Application of strain ML4 fermentation broth in field micro-plots effectively controlled *M. hapla* disease on *A. lappa*, and two applications resulted in superior control efficacy. At 60 days after transplanting, two applications of the undiluted fermentation broth (25.58%) and its 2-fold (44.19%), 5-fold (34.88%), 10-fold (53.49%), and 20-fold (62.79%) dilutions showed better efficacy than 10% fosthiazate. At 90 days after transplanting, two applications of the undiluted fermentation (58.82%), 2-fold (45.1%), 5-fold (54.25%), and 10-fold (49.67%) dilutions were superior to fosthiazate (35.95%), while the 20-fold dilution (31.37%) exhibited efficacy comparable to that of 10% fosthiazate. At harvest, all tested concentrations showed better control efficacy than 10% fosthiazate granules, with the 5-fold dilution (31.96%) achieving the highest relative control efficacy (**Figure 3A**).

#### 3.3.2 SPAD value of *A. lappa*

SPAD measurements at 60 days after transplanting showed that application of high concentrations of the fermentation broth significantly promoted leaf chlorophyll accumulation. At 0&30 d, application of the undiluted fermentation broth (SPAD 43.94), 2-fold (41.78), and 20-fold (43.68) dilutions significantly increased the leaf SPAD value compared with the sterile water control (37.88) and 10% fosthiazate granules (35.83) (p < 0.05). At 0 d, application of the undiluted fermentation (47.64), 2-fold (43.65), 5-fold (41.69), and 20-fold (41.62) dilutions significantly increased the leaf SPAD value compared with the sterile water control (37.88) (p < 0.05) (**Figure 3B**).

#### 3.3.3 Aboveground/underground biomass of *A. lappa*

Biomass investigation at 90 days after transplanting showed that application of different concentrations of the fermentation broth increased the aboveground and underground biomass of *A. lappa* to varying degrees. Application of 10% fosthiazate granules reduced the aboveground and underground biomass accumulation of *A. lappa*, with a relative reduction of 2.29% after a single application and 4.35% after two applications. For the fermentation broth treatments, a single application of the undiluted fermentation (relative increase: 28.92%), and the 10-fold (39.88%), 20-fold (37.63%), 50-fold (14.19%), and 100-fold (35.30%) dilutions, as well as two applications of the undiluted fermentation (4.86%), 20-fold (23.64%), 50-fold (15.42%), and 100-fold (12.25%) dilutions, all increased the underground biomass accumulation of *A. lappa* (**Figure 3C**).

#### 3.3.4 Yield of *A. lappa*

Yield measurement at harvest showed that application of the fermentation broth increased the yield of *A. lappa*. Increased yields were achieved by a single application of the undiluted fermentation broth (relative increase rate: 4.46%), as well as its 2-fold (4.76%), 5-fold (2.29%), 20-fold (1.23%), and 100-fold (1.81%) dilutions, and by two applications of the undiluted fermentation (20.56%) and its 5-fold (10.07%), 10-fold (14.12%), 20-fold (11.67%), 50-fold (11.80%), and 100-fold (4.29%) dilutions (**Figure 3D**).

In the Figure, 0 d: a single application at transplanting; 0&30 d: two applications, one at transplanting and another at 30 days after transplanting; 1: the undiluted fermentation broth; 2, 5, 10, 20, 50, 100: 2-, 5-, 10-, 50-, 100-fold diluted fermentation broth; SZL: application of 10% fosthiazate 0.3 g, CK: water control. Different letters indicate significant differences at P<0.05 based on Fisher’s LSD test.

### 3.4 VOCs in the fermentation broth of *S. liquefaciens* strain ML4

**Figure 4:**
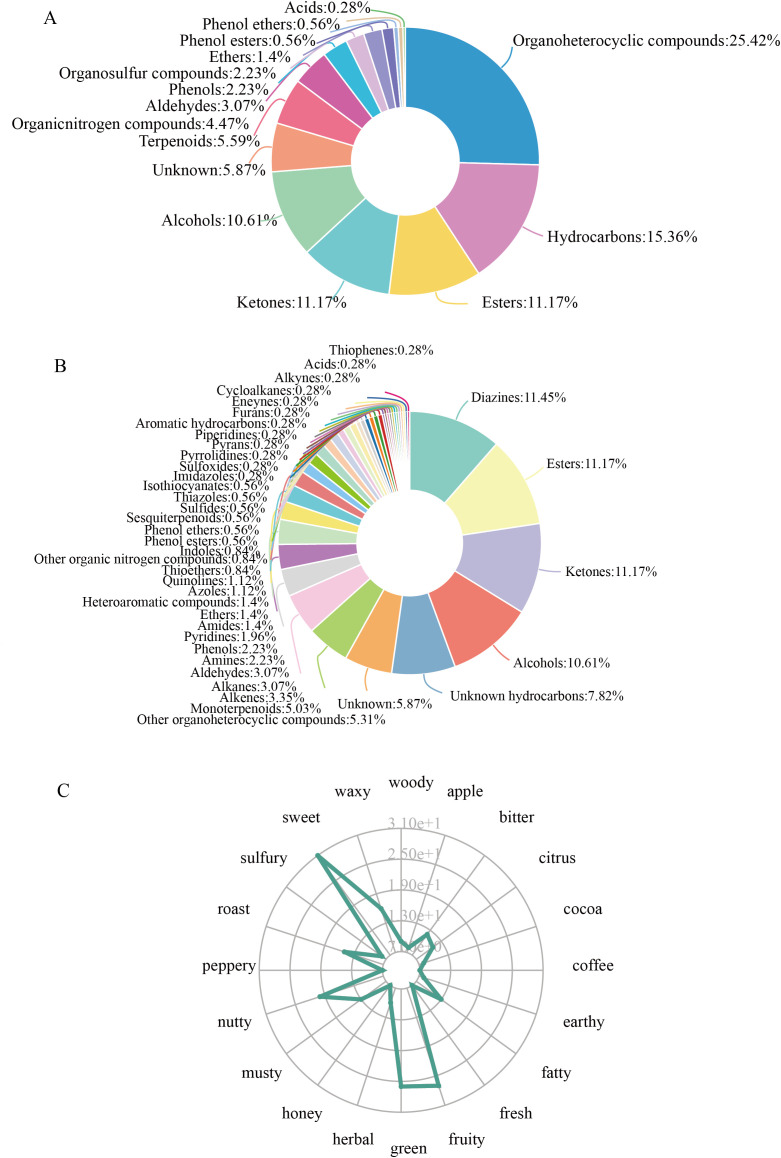
Classification of VOCs in the fermentation broth of *S. liquefaciens*. **(A)** Primary classification of VOCs. **(B)** Secondary classification of VOCs. **(C)** Classification of VOCs based on sensory flavor attributes.

A total of 358 VOCs were detected in the fermentation broth of *S. liquefaciens* ML4 using HS-SPME-GC-MS. These compounds were classified into 15 categories, including organic heterocyclic compounds, hydrocarbons, esters, and ketones (**Figure 4A**), and further into 40 subcategories at the secondary classification level, including alkenes, alkynes, aromatic hydrocarbons, and diazines (**Figure 4B**). A total of 216 sensory flavors were enriched, with the top 20 being sweet, fruity, green, nutty, waxy, roasted, fatty, musty, bitter, citrus, herbal, woody, apple, cocoa, earthy, sulfur, coffee, fresh, honey, and peppery (**Figure 4C**). Odor activity analysis (**Table 1**) revealed that seven compounds significantly contributed to the odor profile of the fermentation broth: 2-sec-butyl-3-methoxypyrazine, 2-isopropyl-3-methoxypyrazine, 1-octen-3-one, 2-isobutyl-3-methoxypyrazine, methyl 2-methyl-3-furyl disulfide, 2,3-butanedione, and dimethyl trisulfide.

**Table 1:** Odor contribution of VOCs in the fermentation broth of *S. liquefaciens*.

Metabolite	ROAV
2-sec-butyl-3-methoxypyrazine	100±0
2-isopropyl-3-methoxypyrazine	24.07±0.08
2-isobutyl-3-methoxypyrazine	24.02±5.49
1-octen-3-one	19.33±0.04
methyl 2-methyl-3-furyl disulfide	18.02±0.32
2,3-butanedione	1.58±0.06
dimethyl trisulfide	1.18±0.03

The relative odor activity value (ROAV) is an important indicator for evaluating the contribution of a compound to the overall odor profile. When ROAV > 1, the compound is considered to make a significant contribution to the odor. ROAV of the sample at 108 h of fermentation. Data are presented as mean ± SD (n = 3).

### 3.5 Fumigation nematicidal activity of six VOCs against *M. hapla*

Based on the distinctive odor of the fermentation broth, VOCs were selected for nematicidal assays against *M. hapla* according to their odor contribution, literature precedence, and application cost. Consequently, six compounds were found to possess nematicidal activity against *M. hapla* (**Table 2**). At a fumigation concentration of 2 μL/L for 24 h, the corrected mortalities were as follows: 1−octen−3−one, 99.06%; 6-methyl-5-hepten-2-one, 96.92%; dimethyl trisulfide, 87.78%; phenylethyl alcohol, 67.03%; dimethyl disulfide, 61.73%; and 2−isobutyl−3−methylpyrazine, 33.15%.

**Table 2:** VOCs with nematicidal activity in the fermentation broth.

VOCs	Corrected mortality
1-octen-3-one	99.06±0.65
6-methyl-5-hepten-2-one	96.92±0.77
dimethyl trisulfide	87.78±6.27
Phenylethyl alcohol	67.03±6.96
dimethyl disulfide	61.73±7.2
2-Isobutyl-3-methylpyrazine	33.15±4.7

Data are presented as mean ± SD (n = 6). Fumigation concentration 2 μL/L, corrected mortality: 24 h corrected mortality of *M. hapla* (%).

### 3.6 Control efficacy of 1-octen-3-one against root-knot nematodes

**Figure 5:**
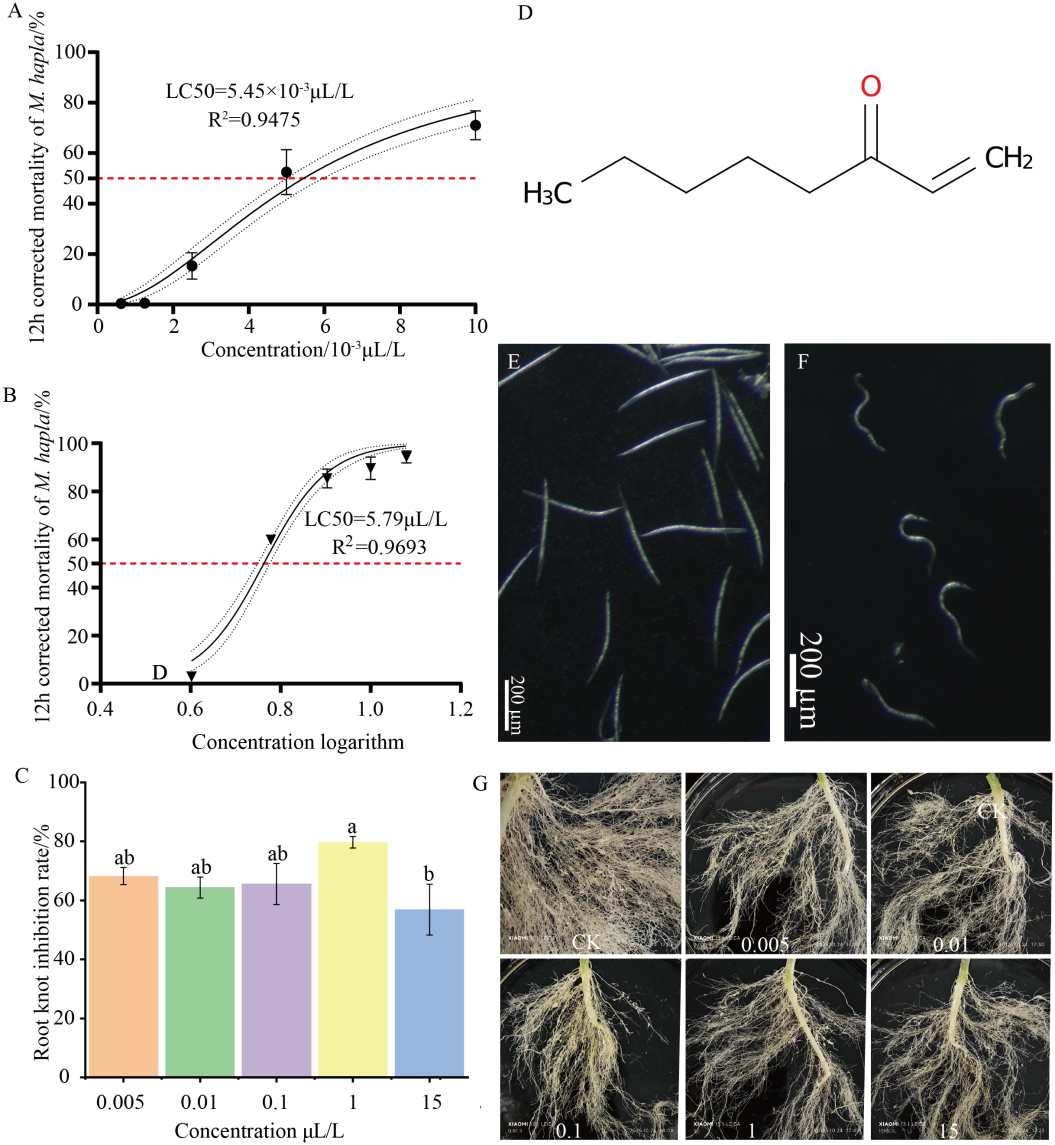
Control efficacy of 1-octen-3-one against root-knot nematodes. **(A)** Fumigation LC_50_ of 1-octen-3-one against *M. hapla* J2s. **(B)** Aqueous contact LC_50_ of 1-octen-3-one against *M. hapla* J2s. **(C)** Gall inhibition rate in pot experiment. **(D)** Chemical structure of 1-octen-3-one. **(E)** Mortality status of *M. hapla* J2s after treatment with 1-octen-3-one. **(F)** *M. hapla* J2s in sterile water control. **(G)** Control efficacy of 1-octen-3-one against root-knot nematode disease on cucumber. In panels **(C, G)**, t the values 0.005, 0.01, 0.1, and 1 represent the concentrations (μL/L soil) of 1-octen-3-one added per liter of soil, while 15 indicates the use of 15 μL/L of 1-octen-3-one aqueous solution.

1-octen-3-one exhibited potent inhibitory activity against *M. hapla*. In the *in vitro* nematicidal assay, the fumigation LC_50_ was 5.45 × 10^-3^ μL/L, while the aqueous contact LC_50_ was 5.79 μL/L, indicating that the fumigation effect was stronger than the contact effect. The pot experiment results were generally consistent with the *in vitro* results. When applied at a concentration of 0.005 μL/L in the soil, the gall inhibition rate was 68.26%; at 0.01 μL/L, the gall inhibition rate was 64.38%; at 0.1 μL/L, it was 65.6%; at 1 μL/L, it was 79.66%; and after treatment with the aqueous solution at 15 μL/L, the gall inhibition rate reached 56.9% (**Figure 5**).

## 4 Discussion

### 4.1 Dual functions of *Serratia* in promoting plant growth and disease control

This study demonstrated that the endophytic strain *S. liquefaciens* ML4 from *A. lappa* effectively controls *M. hapla* and promotes the growth of *A. lappa*. Prior to this, it has been well established that *Serratia* species, as a group of multifaceted plant growth-promoting bacteria (PGPB), possess the multiple functions of disease control, plant growth promotion, and environmental remediation.

Regarding plant growth promotion, *the S. liquefaciens strain UNJFSC 002, isolated from the crop rhizosphere, served* as an inoculant to increase potato yield (Alvarado et al., 2025). The endophytic bacterium *S. marcescens*, isolated from rice stems and roots, exhibited nitrogen-fixing ability and significantly increased root length and root dry weight of rice (Gyaneshwar et al., 2001). *S. plymuthica* strain PN12 possessed ACC deaminase, protease, amylase, pectinase, and cellulase activities, produced IAA and siderophores, exhibited phosphate-solubilizing capacity, and promoted the growth of *Panax notoginseng* (Zhang et al., 2018). Furthermore, *S. liquefaciens* isolated from compost tea produced siderophores, exopolysaccharides, and IAA and acted as a PGPR to promote potato growth (Samet et al., 2022). In the field of environmental remediation, a *S. liquefaciens* strain isolated from the rhizosphere of plants growing in pesticide-contaminated areas tolerated endosulfan at concentrations up to 50,000 mg/L and showed potential to degrade this pesticide (Singh et al., 2022).

In biological control, *Serratia* species exhibit inhibitory activity against a range of pathogens. *S. marcescens* strain B3-SQU showed inhibitory activity against *Pythium aphanidermatum* (Al-Daghari et al., 2023). *S. plymuthica* exhibited inhibitory activity against *M. incognita* on tomato (Zhao et al., 2021). A combination of *S. marcescens* with *Pseudomonas putida* and *P. fluorescens* controlled root-knot nematodes and also showed plant growth-promoting effects (El-Ashry et al., 2021). *S. liquefaciens* effectively controlled *M. incognita* on tomato and increased aboveground biomass (Moslehi et al., 2021).

### 4.2 The production of VOCs by strain ML4 is the key factor in its inhibition of root-knot nematodes

The results indicated that the VOCs produced by *S. liquefaciens* strain ML4, namely 1-octen-3-one, 6-methyl-5-hepten-2-one, dimethyl trisulfide, phenylethyl alcohol, dimethyl disulfide, and 2-isobutyl-3-methylpyrazine, exhibited nematicidal activity against *M. hapla.* This finding revealed the mechanism by which *S. liquefaciens* ML4 controls nematode diseases. Numerous studies have confirmed that specific VOCs are important active factors in bacterial antagonism against plant pathogens. Although the functional diversity of *Serratia* VOCs has been demonstrated across different ecological niches, for example, *S. marcescens* Pt-3 produces the antifungal volatile dimethyl disulfide (Gong et al., 2022), *S. nematodiphila* BC-SKRU-1 produces VOCs that control postharvest green mold of citrus (Boukaew et al., 2024). However, no study has reported on the control of root-knot nematodes by *Serratia* VOCs. The present study is the first to report the nematicidal activity of *S. liquefaciens* ML4 VOCs against *M. hapla*, thus addressing this gap and expanding the functional spectrum of *Serratia* VOCs.

Among the VOCs identified in the fermentation broth of strain ML4, dimethyl disulfide (DMDS) is a particularly notable compound. DMDS is produced by a variety of bacteria, including S. marcescens, *Bacillus cereus*, *B. tropica*, *Burkholderia ambifaria*, *Pseudomonas chlororaphis*, and *P. fluorescens* (Yang et al., 2022). Its mechanisms of action include (i) contact toxicity, which disrupts the nematode body wall structure, causing tissue separation and abnormal vacuole accumulation; and (ii) fumigation activity, which primarily damages the nematode pseudocoelom, leading to organelle vacuolization, mitochondrial swelling, and nuclear membrane rupture (Wang et al., 2023). The contact LC_50_ of DMDS against M. incognita J2s (29.865 mg/L) was approximately 347-fold higher than its fumigation LC_50_ (0.086 mg/L) (Yan et al., 2019), indicating that DMDS exerts its nematicidal effect mainly via fumigation.

In addition, *S. liquefaciens* ML4 also produces 1-octen-3-one, which exhibits potent nematicidal activity. The contact LC_50_ of 1-octen-3-one against *M. hapla* J2s (5.79 μL/L) was approximately 1062-fold higher than its fumigation LC_50_ (5.45 × 10^−3^ μL/L), a pattern highly similar to that observed for DMDS, further confirming that fumigation is the key nematicidal pathway for this compound. This marked difference in activity can be explained by the physicochemical properties of 1-octen-3-one: its high volatility and low water solubility. In the gas phase, it readily penetrates the nematode cuticle and respiratory system, achieving high local concentrations at the target site; in aqueous solution, however, its limited solubility and partitioning at the air–water interface substantially reduce its bioavailability, thereby weakening the contact effect. Therefore, the use of co-solvents to improve the aqueous solubility of 1-octen-3-one should be a focus of future research.

Structurally related compounds, such as 3-octanone and 1-octen-3-ol, have also been investigated for their nematicidal properties. Previous studies have reported that these compounds exhibit potent nematicidal activity against *M. incognita* J2s, and exposure to them leads to a marked increase in intracellular reactive oxygen species (ROS) levels (Kortsinoglou et al., 2026), suggesting that oxidative stress may contribute to their nematicidal effects. Given that both 1-octen-3-one and 6-methyl-5-hepten-2-one exhibit strong fumigation activity, future investigations into their fumigation toxicity mechanisms may draw on the experimental frameworks and detection methodologies previously established for DMDS, 3-octanone, and 1-octen-3-ol.

Notably, this study has confirmed that multiple VOCs, including 1-octen-3-one and 6-methyl-5-hepten-2-one, exhibit nematicidal activity when used individually, indicating that at least part of the nematicidal activity of the *S. liquefaciens* ML4 fermentation broth can be definitively attributed to these compounds. However, the fermentation broth itself is a complex system, and its nematicidal activity is likely not conferred by a single or a few compounds alone. Other unidentified volatile or non-volatile metabolites may exist in the fermentation broth and act synergistically with the target VOCs against nematodes. This does not undermine the core conclusion that VOCs are key contributors to the activity. Given the above complexity, future studies are warranted to systematically evaluate the activity contributions of individual VOCs and their combinations through bioassay-guided fractionation and reconstitution assays to precisely elucidate the potential synergistic mechanisms.

Given the control efficacy of strain ML4 against root-knot nematodes and its growth-promoting effect on A. lappa, the application of this strain can effectively reduce the use of chemical pesticides and fertilizers, demonstrating its potential as a biocontrol agent. Nevertheless, the specific mechanisms underlying its nematicidal activity against root-knot nematodes require further investigation.

## 5 Conclusion

In this study, *Serratia liquefaciens* ML4 was obtained through screening of endophytic bacteria from *Aucklandia lappa.* Through *in vitro* nematicidal assays, pot experiments, and field micro-plot trials, the strain was demonstrated to possess dual functions: controlling root-knot nematode disease and promoting the growth of *A. lappa*. Furthermore, the composition of its fermentation broth was analyzed by HS-SPME-GC-MS, and combined with the results of *in vitro* and pot experiments, we demonstrated that the nematicidal mechanism of this strain primarily depends on its secondary metabolites. In addition, several new VOCs with nematicidal activity, including 1-octen-3-one and 6-methyl-5-hepten-2-one, were discovered from the fermentation broth of strain ML4.

## Data Availability

The raw data supporting the conclusions of this article will be made available by the authors, without undue reservation.
